# Open Source Repurposing Reveals Broad-Spectrum Antiviral Activity of Diphenylureas

**DOI:** 10.3390/v17030385

**Published:** 2025-03-07

**Authors:** Ulrich A. K. Betz, Robert Garces, Norbert Beier, Sven Lindemann, Karen C. Wolff, Laura Riva, Melanie G. Kirkpatrick, Amal Gebara-Lamb, Case W. McNamara, Robert Damoiseaux, Brigitte N. Gomperts, Vaithilingaraja Arumugaswami, Mårten Strand, Yongdae Gwon, Mikael Elofsson, Magnus Evander

**Affiliations:** 1 Merck KGaA, 64293 Darmstadt, Germany; 2 EMD Serono Research & Development Institute, Inc., Billerica, MA 01821, USA; 3 Merck Healthcare KGaA, 64293 Darmstadt, Germany; 4 Calibr, The Scripps Research Institute, 11119 North Torrey Pines Road, La Jolla, CA 92037, USA; 5 Department of Molecular and Medical Pharmacology, University of California, Los Angeles, CA 90095, USA; 6 Umeå Centre for Microbial Research, Umeå University, 901 87 Umeå, Sweden; 7 Department of Clinical Microbiology, Umeå University, 901 87 Umeå, Sweden; 8 Department of Chemistry, Umeå University, 901 87 Umeå, Sweden

**Keywords:** pandemic preparedness, repurposing, broadband antiviral compounds

## Abstract

The pandemic threat from newly emerging viral diseases constitutes a major unsolved issue for global health. Antiviral therapy can play an important role in treating and preventing the spread of unprecedented viral infections. A repository of compounds exhibiting broad-spectrum antiviral activity against a series of different viral families would be an invaluable asset to be prepared for future pandemic threats. Utilizing an open innovation crowd-sourcing paradigm, we were able to identify a compound class of diphenylureas that exhibits in vitro antiviral activity against multiple viruses, including severe acute respiratory syndrome coronavirus 2 (SARS-CoV-2), adenovirus, dengue virus, herpes, and influenza viruses. Compound 4 among the series exhibits strong activity against dengue virus, a growing global health problem with high medical need and no approved antiviral drug. The compounds are active against SARS-CoV-2 in a primary human stem cell-based mucociliary airway epithelium model and also active in vivo, as shown in a murine SARS-CoV-2 infection model. These results demonstrate the potential of the chemical class as antivirals on the one hand and the power of open innovation, crowd-sourcing, and repurposing on the other hand.

## 1. Introduction

Severe acute respiratory syndrome coronavirus 2 (SARS-CoV-2), the virus responsible for the coronavirus disease 2019 (COVID-19) pandemic, since its first emergence in late 2019 in Wuhan, China, has achieved widespread infection of human populations all over the world. Initially, treatment options were limited, but several antiviral drugs have since been developed or repurposed to combat the virus [1,2]. Veklury (remdesivir) was the first antiviral approved by the United States Food and Drug Administration (FDA) for the treatment of COVID-19 and has shown efficacy in shortening recovery times in hospitalized patients but was only available as intravenous formulation. Remdesivir shows high structural similarity to adenosine, as its pharmacologically active triphosphate form inhibits the viral RNA-dependent RNA polymerase. It was initially developed as a potential treatment for hepatitis C virus (HCV). Later, in the COVID-19 pandemic, Lagevrio (molnupiravir) and Paxlovid (a combination of nirmatrelvir and ritonavir) were authorized for emergency use, providing additional long awaited oral antiviral options for treating mild to moderate cases. Molnupiravir (also known as EIDD-2801 or MK-4482) is a prodrug of the ribonucleoside analog N4-hydrocytidine. After being metabolized to its active form within the cell, it is incorporated into the viral RNA by the SARS-CoV2-RNA-dependent RNA polymerase, inducing a high frequency of mutations. Nirmatrelvir is a peptidomimetic inhibitor targeting the viral 3C-like cysteine protease. Ritonavir is a potent inhibitor of cytochrome P450 3A4 (CYP3A4), thereby slowing down the degradation of nirmatrelvir. It was initially developed as an HIV protease inhibitor. Furthermore, ensitrelvir (Xocova), an orally active 3C-like protease inhibitor, achieved emergency approval for treatment of COVID-19 in Japan and olgotrelvir, a Mpro protease inhibitor and cathepsin L inhibitor, reached its clinical endpoints in a phase III study.

Pandemic threats from newly emerging viral diseases pose significant risks to global public health. A recent study analyzing farmed fur animals revealed that they harbor an alarming number of new and so far unknown viruses with zoonotic spillover potential [3]. As seen during the COVID-19 pandemic, such threats can overwhelm healthcare systems, disrupt economies, and strain supply chains, leading to widespread detrimental societal impacts. Factors such as globalization, climate change, and the threat of bioterrorism increase the likelihood of future pandemics with potentially devastating global impact.

A broad-spectrum antiviral drug would be an extremely valuable asset to protect humanity from the spread of newly emerging pandemic diseases. Such a drug, capable of targeting multiple viruses or viral families, could offer rapid treatment options in the face of novel pathogens before targeted treatments or vaccines become available. This would be especially useful in containing outbreaks and reducing the severity of infections, potentially preventing a localized epidemic from becoming a global pandemic. The development of these drugs would revolutionize pandemic preparedness and greatly enhance global health security.

While some drugs exhibit limited activity against multiple viruses, a convincing broad-spectrum antiviral drug does not yet exist. For example, remdesivir has been used against Ebola and COVID-19, while ribavirin is effective against both HCV and respiratory syncytial virus (RSV). Most antivirals are tailored against specific viruses, such as acyclovir for herpesviruses or oseltamivir for influenza. A broad-spectrum antiviral would have to interfere with the replication of highly diverse phylogenetically unrelated viral species, which is challenging via targeting viral proteins but could potentially be achieved via addressing cellular target pathways that are required for the replication of multiple virus families. Detailed investigation of the replication of SARS-CoV-2, for example, has revealed several potentially suitable pathways [2,4,5]. Ongoing research continues to search for more universal antiviral therapies, but achieving the goal remains elusive.

Open innovation and crowd-sourcing approaches have been demonstrated as valuable modules to generate innovation also in chemistry and pharmacology [6].

Leveraging this insight, in order to increase the knowledge gathered for former developmental or advanced research compounds for the potential future benefit of patients, the pharmaceutical company Merck KGaA, Darmstadt, Germany, decided to provide a set of 80 de-prioritized research compounds to interested researchers. The goal is to assess these compounds in as many as possible new indications, therapeutic areas, and assays to identify potential new actions and, thus, potential new therapeutic utility. The set of 80 compounds, coined the Merck Mini Library, comprises representative structures of about 20 different pharmacological classes; the number of compounds per pharmacological class vary between one and up to eight. The Mini Library is provided upon request to the external party free of charge. A comprehensive summary of the data collected by Merck KGaA, Darmstadt, Germany, for these structures is provided together with the 96-well microtiter plate, which contains the eighty 50 µL of the 10 mM DMSO solutions of the respective compounds [7]. The distribution of the unique set of compounds has been in place since 2014 and has already resulted in the identification of compounds for the treatment of Cryptosporidium parvum [8].

Here, we describe the identification of a compound class of diphenylureas with promising broad-spectrum antiviral activity in vitro and in vivo uncovered via repurposing activities using this library.

## 2. Materials and Methods

### 2.1. Primary Antiviral Screening

The Mini Library was screened in a dose–response manner against human adenovirus (HAdV) and Rift Valley fever virus (RVFV) using a replication–competent green fluorescent protein (GFP)-expressing HAdV vector based on serotype 11 (RCAd11pGFP) at a concentration of 0.125 pg virus/cell [9]. and a far-red fluorescent protein-expressing RVFV vector (rRVFVDNSs::Katushka) at a multiplicity of infection (MOI) of 0.3 [10]. The Mini Library was serially diluted in dimethylsulfoxide (DMSO) to reach a final concentration span starting from 100 μM down to 0.4 μM (9 two-fold steps). Benzavir-2, a preclinical compound with activity against HAdV [11] and RVFV [12], was used as positive control. On the day before infection, 12,000 A549 cells/well were seeded in black 96-well plates with a clear bottom (Greiner Bio-One International, Kremsmünster, Austria) using Dulbecco’s Modified Eagle’s Medium (DMEM, Sigma-Aldrich, St. Louis, MO, USA) containing 0.75 g NaHCO_3_/L, 20 mM 4-(2-hydroxyethyl)-1-piperazineethanesulfonic acid (HEPES, EuroClone, Milan, Italy), 1× PEST (penicillin G, 100 IU/mL, and streptomycin sulfate, 100 μg/mL, Gibco, Carlsbad, CA, USA), and 5% fetal bovine serum (FBS, Gibco) at 37 °C. The day after, the compounds were diluted in DMSO to reach the final compound concentration in the well volume (100 μM/well). For the infection, the virus was diluted in cell media (as described above but with 2% FBS) and added to the cells together with the compounds. Sixteen h post infection, the RVFV-infected cells were fixed with 4% pbs-buffered paraformaldehyde for 30 min and the wells were washed 3 times with phosphate-buffered saline (PBS) and 250 μL PBS was added prior to fluorescence analysis. For the HAdV-infected cells, the wells were washed three times in PBS 24 h post infection and the wells were filled with 250 μL PBS prior to analysis. The viral specific fluorescence analysis was performed using Trophos plate runner HD (Trophos, Roche Group, Marseille, France) at 485 nm for HAdV and 588 nm for RVFV. Additionally, visual observation of the cells was performed prior to the analysis to identify compound toxicity and/or precipitation.

### 2.2. SARS-CoV-2 Plaque Reduction Assay

VeroE6 cells (5 × 10^5^/well) were seeded in 12-well plates 24 h before infection, and 250 plaque-forming units per well (pfu/well) of SARS-CoV-2 (SARS-CoV-2/human/SWE/01/2020) together with a 3-fold serial dilution of compounds **1**, **2**, and **3** was added. After 1 h, 2 mL semisolid overlay containing DMEM  +  2% FBS  +  PEST  + 2% carboxymethyl cellulose (CMC) and the compound was added, and the cells were incubated at 37 °C in 5% CO_2_. After 65 h, the overlay was removed and the cells were fixed with 4% formaldehyde for 30 min, washed with PBS, and stained with 0.5% crystal violet in 20% MeOH for 5 min. The plates were washed with water and the plaques were counted.

### 2.3. SARS-CoV-2/HeLa-ACE2 High-Content Screening Assay

The compounds were acoustically transferred into 384-well clear-bottom plates (Greiner, Part. No. 781090-2B). HeLa-ACE2 cells were seeded in 13 µL DMEM with 2% FBS at a density of 1.0 × 10^3^ cells per well. The plated cells were transported to the BSL3 facility where 13 µL of SARS-CoV-2 (strain USA-WA1/2020 propagated in Vero E6 cells) diluted in assay media was added per well at a concentration of 2.0 × 10^6^ PFU/mL (assay multiplicity of infection (MOI) = 0.65). The plates were incubated for 24 h at 34 °C 5% CO_2_ and then fixed with 25 µL of 8% formaldehyde for 1 h at 34 °C 5% CO_2_. The plates were washed with 1× PBS 0.05% Tween 20 in between fixation and subsequent primary and secondary antibody staining. Human polyclonal sera diluted 1:500 in Perm/Wash buffer (BD Biosciences 554723) was added to the plate and incubated at room temperature for 2 h. Six µg/mL of goat anti-human H + L conjugated Alexa 488 (Thermo Fisher Scientific A11013) together with 8 µM of antifade-46-diamidino-2-phenylindole (DAPI; Thermo Fisher Scientific D1306) in SuperBlock T20 (PBS) buffer (Thermo Fisher Scientific 37515) was added to the plate and incubated at room temperature for 1 h in the dark. The plates were imaged using the ImageXpress Micro Confocal High-Content Imaging System (Molecular Devices) with a 10× objective, with 4 fields imaged per well. The images were analyzed using the Multi-Wavelength Cell Scoring Application Module (MetaXpress), with DAPI staining identifying the host–cell nuclei (the total number of cells in the images) and the SARS-CoV-2 immunofluorescence signal leading to the identification of infected cells.

#### 2.3.1. Uninfected Host Cell Cytotoxicity Counter Screen

The compounds were acoustically transferred into 1536-well µclear plates (Greiner Part. No. 789091). HeLa-ACE2 cells were maintained as described for the infection assay and seeded in the assay-ready plates at 400 cells/well in DMEM with 2% FBS and the plates were incubated for 24 h at 37 °C 5% CO_2_. To assess cell viability, the Image-iT DEAD green reagent (Thermo Fisher, Waltham, MA, USA) was used according to the manufacturer’s instructions. The cells were fixed with 4% paraformaldehyde and counterstained with DAPI. The fixed cells were imaged using the ImageXpress Micro Confocal High-Content Imaging System (Molecular Devices, San Jose, CA, USA) with a 10× objective, and the total live cells per well were quantified in the acquired images using the Live Dead Application Module (MetaXpress).

#### 2.3.2. Data Analysis

Image analysis was carried out with MetaXpress (version 6.5.4.532). Primary in vitro screen and the host cell cytotoxicity counter screen data were uploaded to Genedata Screener, Version 16.0.3-Standard. The data were normalized to neutral (DMSO) minus inhibitor controls (2.5 µM remdesivir for antiviral effect and 10 µM puromycin for infected host cell toxicity). For the uninfected host cell cytotoxicity counter screen 40 µM puromycin (Sigma) was used as the positive control. For dose response experiments, the compounds were tested in technical triplicates on different assay plates and the dose curves were fitted with the four parameter Hill Equation. Technical replicate data were analyzed using median condensing.

### 2.4. SARS-CoV-2/Calu-3 High-Content Screening Assay

The compounds are acoustically transferred into 384-well µclear-bottom plates (Greiner, Part. No. 781090-2B) before seeding Calu-3 cells in assay media (MEM with 2% FBS) at a density of 5000 cells per 20 μL per well. The plated cells are transported to the BSL3 facility where SARS-CoV-2 (strain USA-WA1/2020 propagated in Vero E6 cells) diluted in assay media is added at an MOI between 0.75 and 1 to achieve ~30–60% infected cells. The plates are incubated for 48 h at 34 °C 5% CO_2_ and then fixed with a final concentration of 4% formaldehyde. The fixed cells are stained with human polyclonal sera as the primary antibody, goat anti-human H + L conjugated Alexa 488 (Thermo Fisher Scientific A11013) as the secondary antibody, and antifade-46-diamidino-2-phenylindole (DAPI; Thermo Fisher Scientific D1306) to stain DNA, with PBS 0.05% Tween 20 washes in between fixation and subsequent primary and secondary antibody staining.

The plates are imaged using the ImageXpress Micro Confocal High-Content Imaging System (Molecular Devices) with a 10× objective, with 4 fields imaged per well. The images are analyzed using the Multi-Wavelength Cell Scoring Application Module (MetaXpress), with DAPI staining identifying the host–cell nuclei (the total number of cells in the images) and the SARS-CoV-2 immunofluorescence signal leading to the identification of infected cells.

#### 2.4.1. Uninfected Host Cell Cytotoxicity Counter Screen

The compounds are acoustically transferred into 1536-well plates (Corning No. 9006BC) before seeding Calu-3 cells in assay media (MEM with 2% FBS) at a density of 600 cells per 5 μL per well. The plates are incubated for 48 h at 37 °C 5% CO_2_. To assess cell viability, 2 μL of 50% Cell-Titer Glo (Promega No G7573) diluted in water is added to the cells and luminescence is measured on an EnVision Plate Reader (Perkin Elmer, Waltham, MA, USA).

#### 2.4.2. Data Analysis

Data from the SARS-CoV-2 antiviral assay and host cell cytotoxicity counter screen are uploaded to Genedata Screener, Version 16.0. For the SARS-CoV-2 antiviral readout, the % CoV-2 positive cells are normalized to neutral (DMSO) minus inhibitor controls (10 µM remdesivir). For the cell count readout, the total cells are normalized to the stimulator (10 µM remdesivir) minus the neutral control (DMSO). The uninfected host cell cytotoxicity counter screen is normalized to neutral (DMSO) minus the inhibitor control (30 µM puromycin). For the dose response experiments, the compounds are tested in technical triplicates on different assay plates and the dose curves are fitted with the four parameter Hill Equation. The curves are fitted as either increasing or decreasing and noted as such in the data output. This is of particular note for the cell count readout from the SARS-CoV-2 infection assay, which captures an antiviral effect, protection from virus-induced cell death (increasing), and cellular toxicity (decreasing).

### 2.5. Broad-Spectrum Antiviral Profiling

Broad-spectrum antiviral profiling was performed at IBT Bioservices using the following strains: adenovirus 5 (HAdV5), chikungunya virus (CHKV) 181/25, dengue virus (DNV) serotype 2 D2Y98P, herpes simplex virus (HSV) 1 MacIntyre, Herpes simplex virus (HSV) 2 MS, influenza virus (INFV) H1N1 A/California/07/09, and Zika virus (ZIKV) FSS13025. A total of 10,000 Vero cells were seeded per well in 96-well flat bottom tissue culture plates in growth medium (MEM supplemented with 10% HI-FBS, P/S and L-Gln) and incubated overnight at 37 °C and 5%CO_2_. Eight 5-fold serial dilutions of each test article (TA) were prepared in serum-free medium at two times (2×) the final intended concentration; for INFV only, 1 µg/mL of TPCK trypsin was added to the medium. Growth medium from the 96-well plates was then removed; 50 µL fresh serum-free medium was added to each well followed by 50 µL of each TA dilutions in triplicates; the cell only and virus only wells received 100 µL of medium only. The plates were incubated at 35 °C (for INFV only) or 37 °C and 5% CO_2_ for 60 min ± 5 min.

Each virus strain was prepared in serum-free medium at a specific MOI of 0.01; for INFV only, 1 µg/mL of TPCK trypsin was added to the medium. A total of 100 µL of virus inoculum was then mixed to 100 µL of each TA concentration, including virus only wells; 100 µL of the medium only was also added to the cell only wells for a final 200 µL per well. The plates were incubated at 35 °C (for INFV only) or 37 °C and 5% CO_2_ for a different number of days before cell fixation and staining.

After incubation, the media were removed and the cells were fixed with cold 80%/20% (*v*/*v*) ethanol/methanol and incubated for 20 min at −20 °C. The cells were washed 3× with 1× DPBS and 200 µL of the diluted anti-virus antibody (1:2000 in blocking buffer) was added to each well for overnight incubation at 4 °C (Anti-INFV A antibody (mAb), EMD Millipore, MB8251; Purified Mouse Anti-Adenovirus (mAb), BD Pharmingen, 554155; Anti-DENV antibody (mAb), 4G2, IBT). The primary antibody was removed and the plates were washed three times with 1× DPBS. The common secondary antibody, Goat Anti-Mouse IgG (H + L)-HRP Conjugate diluted 1:2000 in blocking buffer, was then added at 200 µL/well and the plates were incubated for 1 h at room temperature. After removing the secondary antibody, the plates were washed three times with 1× DPBS. TMB substrate (150 µL/well) was added for approximately 10 min (or until the virus only wells were intensely stained while the cell only wells were still weak in signal). Stop solution was then added to each well and optical density was read at 450 nm.

After incubation, the media were removed and the cells were fixed and stained with 0.1% crystal violet solution (0.05% methanol, 50% Glutaric dialdehyde in 1× DPBS) for 60 min ± 5 min at room temperature. The cells were washed 3× with 1× DPBS and after removing all liquid, the returned plates were air dried at room temperature. Optical density was read at 570 nm for each well.

Similar cell and TA preparation was conducted in parallel for this assay to mimic as closely as possible the antiviral assay. Virus inoculum was replaced by serum-free medium only, and for INFV only, 1 µg/mL of TPCK trypsin was added to the medium. To cover all the different incubation times with only 2 conditions, the plates were incubated at 35 °C (for INFV only) or 37 °C and 5% CO_2_ for 3 and 5 days.

All media were removed and 100 µL of DPBS was added to each, followed by 100 µL of CellTiter-Glo^®^ reagent. The plates were gently shaken for 2 min and incubated at RT for 10 min before determining luminescence.

All data were imported into Excel. The XLfit 5 plug-in was used with fit# 205 (Levenberg–Marquardt algorithm) to determine the 50 percent inhibition concentration (IC50) and the 50 percent cytotoxicity concentration (CC_50_).

Further broad-spectrum antiviral profiling was performed at Virology Research Services using human foreskin fibroblasts (HFFs), passage 3 and human cytomegalovirus (HCMV) Merlin strain, passage 2 (National Collection of Pathogenic Viruses, Public Health England). The antiviral activity of 8 dilutions of each compound was explored following administration 1 h before infection with HCMV. The compound and virus were left on the cells for the entire duration of the experiment (5 days). The cytotoxicity of the same range of concentrations of the compounds was determined by MTT assay. The cells were detached and counted. The cells were seeded in complete media at 4000 cells/100 µL/well in four 96-well plates: two for the cytotoxicity assay and two for the infectivity assay. After seeding, the plates were incubated at RT for 5 min for even distribution, and then at 37 °C, 5% CO_2_ until the following day. The test compound stocks (10 mM) were diluted to 200 µM in supplemented media, and 225 µL of these diluted stocks or diluent only (1% DMSO) were added in triplicate to the top row (A) of a round bottom 96-well plate. A total of 180 µL of 0.2% DMSO diluent were added in all other wells (rows B-H). In this way, the percentage of DMSO was kept constant at 0.2% across the serial dilution. Only in row A the concentration of DMSO was 1% (also in the uninfected/untreated controls), reflecting the DMSO concentration in the first dilution from the stock. A five-fold serial dilution was performed by transferring 45 µL from row A into row B, mixing, and then again from row B into C, etc., until row H. A total of 50 µL of the supplemented media per well was added to the cells in each plate (infectivity and cytotoxicity). A total of 50 µL per well of treatment from the dilution plate was transferred to the cells in corresponding positions in each plate (infectivity and cytotoxicity). All plates were incubated at 37 °C, 5% CO_2_. The virus stock (HCMV Merlin strain, 1 × 10^6^ IU/mL) was diluted 5-fold with the supplemented media to bring the concentration to 2 × 10^5^ IU/mL. After 1 h pre-treatment, the media/treatment was removed from the cells and 50 µL per well of treatment from the dilution plate was re-transferred to the cells in corresponding positions in the infectivity plates. A total of 50 µL virus per well (MOI ~1) wwasere added, except in column 11 (uninfected control), where 50 µL of the supplemented media without virus was added. After five days, the infected plates were washed with PBS, fixed for 30 min with 4% formaldehyde, washed again with PBS, and stored in PBS at 4 °C overnight until staining. The cytotoxicity plates were treated with MTT to determine cell viability. The cells were immunostained following SOP-RA 005. Briefly, any residual formaldehyde was quenched with 50 mM ammonium chloride, after which the cells were permeabilized (0.1% Triton X100) and stained with an antibody-recognizing HCMV gB (The Native Antigen Company, Kidlington, UK). The primary antibody was detected with an Alexa-488 conjugate secondary antibody (Life Technologies, Waltham, MA, USA, A21207), and the nuclei were stained with Hoechst. The images were acquired on a CellInsight CX5 high-content confocal microscope (Thermo Fisher, Waltham, MA, USA) using a 4X objective, and percentage infection was calculated using the CX5 software (infected cells/total cells × 100). Cytotoxicity was detected by MTT assay following SOP-RA 006. Briefly, the MTT reagent (Sigma, M5655) was added to the cells for 2 h at 37 °C, 5% CO_2_, after which the media was removed and the precipitate solubilized with a mixture of 1:1 Isopropanol/DMSO for 20 min. The supernatant was transferred to a clean plate and signal read at 570 nm. The normalized percentages of the inhibition were calculated.

EC50 values were extrapolated from the curves representing the best fit (non-linear regression analysis, variable slope) of the logarithm of the compound concentrations vs. the normalized percentages of the inhibition, using GraphPad Prism (version 9). The percentages of cytotoxicity were calculated.

### 2.6. Human Tissue Procurement

Large airways and bronchial tissues were acquired from three different tissue sources: (1) de-20 identified normal human donors after lung transplantations at the Ronald Reagan UCLA Medical Center. Tissues were procured under Institutional Review Board-approved protocols at the David Geffen School of Medicine at UCLA. (2) Normal human bronchial epithelial cells (NHBE) from non-smokers were obtained from Lonza and all samples were de-identified. (3) IIAM donor lung and trachea samples were obtained with institutional approval.

### 2.7. Airway Basal Stem Cell (ABSC) Isolation

Human ABSCs were isolated with all steps performed with the trachea in cold PBS with antimicrobials. Briefly, proximal cartilaginous airways were dissected, cleaned, and incubated in 50 U/mL dispase for 45 min at room temperature. The tissues were then incubated in 0.5 mg/mL DNase for another 45 min at room temperature. Epithelium was stripped and scraped with a cell-scraper and incubated in 0.25% Trypsin-EDTA for 30 min with shaking at 37 °C to generate a single cell suspension. The isolated cells were passed through a series of strainers, 500 μm, 300 μm, and 100 μm, and either plated directly (Passage 0 (P0)) for Air Liquid Interface (ALI) cultures or expanded in collagen IV (0.5 mg/mL) coated T25 or T75 up to P1/P2 and then plated for ALI cultures.

### 2.8. 96-TW ALI Cultures

Moreover, 96-TW with 0.4 μm pore polyester membrane inserts (Corning 7369) were coated with 0.5 mg/mL collagen type IV and allowed to air dry overnight inside a biosafety cabinet. The ABSCs (P0–P2) were seeded at 8000–10,000 cells per well onto collagen-coated transwells in Pneumacult Ex Plus media 21 (STEMCELL Technologies, Cambridge, UK), supplemented with Primocin (Invivogen, San Diego, CA, USA) and 1× Penicillin-Streptomycin-Neomycin (PSN) Antibiotic Mixture (Thermo Fisher Scientific). The ABSCs were allowed to grow in the submerged phase with 180 μL media in the basal chamber and 75 μL media in the apical chamber until they were 80–100% confluent and tight cell junctions were formed (5–7 days). The ALI cultures were then established via airlifting, and the cells were cultured with 180 μL Pneumacult ALI (STEMCELL technologies) media only in the basal chamber for 21 days until viral infection. The media were changed every other day and the cultures were maintained at 37 °C and 5% CO_2_. In the case of drug testing, the drugs were added at their required concentration in the basal chamber media 24 h before viral infection.

### 2.9. SARS-CoV-2 Infection in ALI Cultures

SARS-CoV-2 strains including Isolate USA-WA1/2020 (Parental, BEI Resources NR-52281) at a low MOI (0.1), followed by the Isolate hCoV-19/USA/MD-HP01542/2021 (Beta, BEI Resources NR-55282), Isolate hCoV-19/USA/MD-HP05647/2021 (Delta, BEI Resources NR-55672), and Isolate hCoV-19/USA/MD-HP20874/2021 (Omicron, BEI Resources NR-56461), were obtained from Biodefense and Emerging Infectious (BEI) Resources of the National Institute of Allergy and Infectious Diseases (NIAID). All the studies involving live viruses were conducted in the UCLA BSL3 high-containment facility with appropriate institutional biosafety approvals. SARS-CoV-2 was passaged once in Vero-E6 cells and viral stocks were aliquoted and stored at −80 °C. Virus titer was measured in Vero-E6 cells by TCID50 assay.

The ALI cultures on the apical chamber of the transwell inserts were infected with SARS-CoV-2 viral inoculum (MOI of 0.1; 100 μL/well) prepared in PneumaCult ALI (STEMCELL technologies) media. The basal chamber of the transwell contained 180 μL of ALI media. For mock infection, ALI media (30 μL/well) alone was added. The inoculated plates were incubated for 2 h at 37 °C with 5% CO_2_. At the end of incubation, the inoculum was removed from the apical chamber. At selected time points, live cell images were obtained by bright field microscopy to detect the cytopathic effect (CPE) in SARS-CoV-2-infected cells, indicating viral replication and associated cell injury. At 72 h post-infection (hpi), the ALI cultures were fixed in 4% paraformaldehyde followed by immunofluorescence (IF) analysis.

### 2.10. Nanoluciferase

We used the Nano-Glo Luciferase Assay System as indicated by the manufacturer for assessing the presence of luciferase activity. The ALI cultures were pre-treated with drug compounds for 24 h. The pre-treated ALI cultures were then infected with SARS-related coronavirus 2, Isolate USA-WA1/2020 (icSARS-CoV-2-nLuc) (BEI Resources NR-54003). After 48 hpi, a working reagent luciferin (100 μL) was added to the cells and incubated for at least 3 min at room temperature. Thereafter, the luminescence of each condition was measured in triplicate values and recorded. Percent viability for each compound was calculated based on the vehicle (water or DMSO)-treated cells.

### 2.11. Immunofluorescence and Confocal Imaging

Post fixation with 4% PFA, the ALI cultures were washed 3 times with Tris-Buffered Saline and Tween-20 (TBST) followed by permeabilization with 0.5% Triton-X for 10 min. The ALIs were then blocked using serum-free protein block (Dako X090930) for 1 h at room temperature and overnight for primary antibody incubation. Based on preliminary trials, we optimized different SARS-CoV-2 antibodies for different strains of the SARS-CoV-2 virus.

The following were used for the various strains: NR-10361 Polyclonal Anti-SARS Coronavirus (antiserum, Guinea Pig) for the Wuhan and Beta strains; SARS-CoV-2 [(2019-nCoV) Spike S1 Antibody, Rabbit Mab] for the Delta strain; and SARS-CoV-2 nucleocapsid antibody (GeneTex, GTX135357) for the Omicron strain. After several washes with TBST, the secondary antibodies were incubated on samples for 1 h in darkness, washed, and used for confocal imaging. High-throughput, high-content confocal images were obtained using ImageXpress (Molecular Devices) with a 20× objective and the number of infection clusters per well were quantified by a developed algorithm. To compare ALI differentiation in 24- and 96-TW, images were captured on Zeiss LSM 880 confocal microscopes.

### 2.12. Image Quantification

The plates were imaged using a Molecular Devices Confocal Imager at 20× resolution with an extra long working distance (ELWD) objective (n/a = 0.45) with at least 14 fields per well on an Image Xpress Confocal (Molecular Devices) while avoiding the corners of the well. Each site in each well was individually focused by images for the DAPI channel and pictures obtained for both the DAPI and TRITC channels. The images were streamed real time into an SQL managed instance of MetaXpress Software (version 6.5) with a dedicated database and file server, respectively. The image analysis workstation used for data analysis was running ImageXpress equipped with Custom Module Editor. The DAPI channel was utilized to ensure an even cell layer without any structural defects and to identify any compounds with gross toxicity effects via visual inspection. To detect clusters of cells infected with SARS-CoV-19, we used an adaptive thresholding approach. Objects ranging from 21.9 to 250 µm with a brightness of 4500 grayscales over local background were scored as positive and the number of infected cell clusters as well as average intensity was recorded. The average number of infected cell clusters was determined for each well as the mean of all sites for each well.

### 2.13. In Vivo Mouse Studies of SARS-CoV-2 Infection

In vivo experiments were approved by the institutional animal welfare study group and performed at the CRO Vibiosphen, Labège, France. At day 0, the mice (B6.Cg-Tg(K18-ACE2)2Prlmn/J males, 7-week-old) were pre-treated by oral gavage 1 h before infection (100 mg/kg compound in 0.5% Methocel/0.25% Tween-20/water). At day 0 + 1 h, the mice were infected with 25 µL of DMEM containing SARS-Cov-2 (1 × 10^4^ PFU/mouse) through the intranasal route. From day 0 to day 5, the mice were treated by oral gavage twice daily. The pause between treatments was set to 12 h. At day 1, day 3, and day 6, 5 mice from each group were euthanized by cervical dislocation for the collection of lung tissue and virus quantification.

Lung tissues (up to 30 mg) were homogenized in RLT buffer and RNA was extracted using the RNeasy kit (Qiagen, Germantown, MD, USA) according to the manufacturer’s instructions. Viral RNA was detected by qRT-PCR, as described in [13].

A 25 μL reaction contained 5 μL of RNA, 12.5 μL of 2 × reaction buffer provided with the Superscript III one step RT-PCR system with Platinum Taq Polymerase (Invitrogen, Darmstadt, Germany; containing 0.4 mM of each deoxyribont triphosphates (dNTP) and 3.2 mM magnesium sulfate), 1 μL of reverse transcriptase/Taq mixture from the kit, 0.4 μL of a 50 mM magnesium sulfate solution (Invitrogen), and 1 μg of nonacetylated bovine serum albumin (Roche, Marseille, France). All oligonucleotides were synthesized and provided by Tib-Molbiol (Berlin, Germany). Thermal cycling was performed at 55 °C for 10 min for reverse transcription followed by 95 °C for 3 min and then 45 cycles of 95 °C for 15 s, 58 °C for 30 s. Participating laboratories used either Roche Light Cycler 480II or Applied Biosystems ViiA7 instruments (Applied Biosystems, Hong Kong, China). Ten-fold dilutions of SARS-CoV-2 standards with known copy numbers were used to construct a standard curve.

## 3. Results

The Merck Mini Library consists of a selection of legacy development compounds and compounds from advanced research projects, which had been de-prioritized during strategy changes. The molecular targets comprise enzymes (e.g., kinases, phosphatases, proteases, cyclic nucleotide phosphodiesterases (PDEs), etc.), hormone or neurotransmitter receptors (serotonin, angiotensin II, endothelin, etc.), transporters, ion channels, and several others (Table A1). For most of these compounds and target classes, the originally selected structures were complemented by additional, structurally related ones, which would allow for a first, rough assessment of a structure–activity relation (SAR) in case of a screening hit. All compounds are small organic compounds supplied in 50 µL of a 10 mM solution in dimethyl sulfoxide (DMSO) on a 96-well microtiter plate.

The Merck Mini Library was screened in a full dose–response manner against both human adenovirus (HAdV) and Rift Valley fever virus (RVFV) in A549 cells using fluorescent reporter-expressing vectors. HAdVs are non-enveloped double-stranded DNA viruses that infect humans, resulting in a wide variety of symptoms [14]. In otherwise healthy individuals, the infections are mostly self-limiting but in immunocompromised patients, mortality rates can be high. RVFV on the other hand is an enveloped zoonotic RNA virus causing the disease Rift Valley fever that results in significant morbidity in both humans and live-stock, predominantly in Africa [15]. Both viruses lack specific approved drugs for the treatment of infected humans. We argued that a compound showing antiviral activity against the two different viruses holds promise for further development into broad-spectrum host-directed antivirals. Several compounds in the Mini Library had a potent antiviral effect against both or one of the viruses and in many cases, this effect was also associated with host cell toxicity. Based on the overall screening results, we selected the three Tie-2 annotated compounds for further investigation.

Compounds **1**–**3** were included in the library due to vanadate-induced inhibition of TIE-2 receptor phosphorylation in Chinese hamster ovary (CHO) cells with an IC_50_ = 27 nM (compound **1**), 3 nm (compound **2**), and 36 nM (compound **3**).

**Figure 1:**
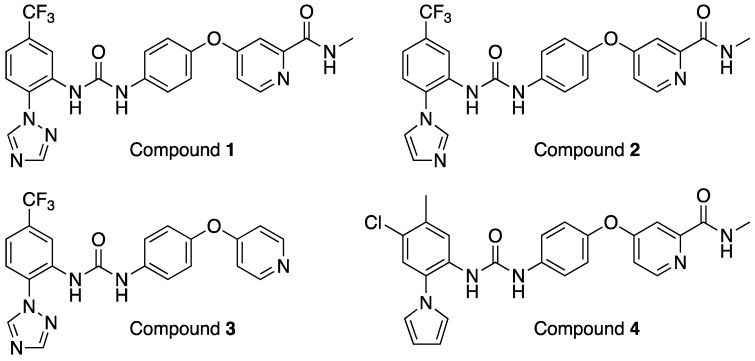
Structures of TIE-2 inhibitors compound **1**–**4**.

**Figure 2:**
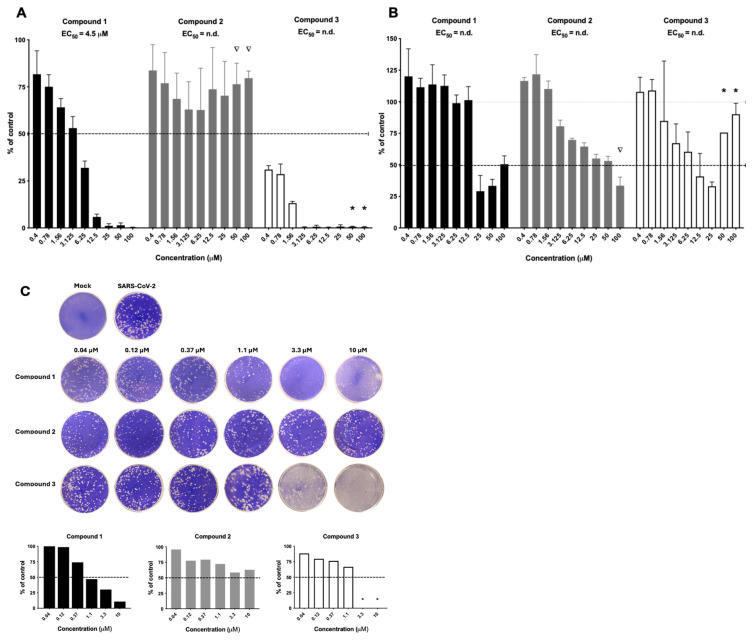
The dose–response activity of the three TIE-2 inhibitors from the Merck Mini Library. The results from the primary screening are displayed in (**A**) against HAdV and (**B**) against RVFV in A549 cells. Ñ = visual compound precipitation; * = visual host cell toxicity. (**C**) Activity against SARS-CoV-2 in VeroE6 cells using plaque reduction assay. * = visual host cell toxicity.

From the primary screening, compounds **1**, **2**, and **3** (Figure 1) displayed an intriguing profile against both HAdV (Figure 2A) and RVFV (Figure 2B). Compound **1** efficiently inhibited HAdV in a clear dose–response relationship with an EC_50_ value of 4.5 µM. For RVFV, antiviral activity was seen at 25 µM but an EC_50_ value could not be calculated due to invalid curve fit. Interestingly, compound **2** did not have activity against HAdV but did have dose–response antiviral activity against RVFV, albeit no robust EC_50_ value could be generated. Compound **3** was the most potent compound but displayed host cell toxicity at the highest concentrations, as assessed by visual observation of the cells in the primary screening.

Considering the ongoing health issues during the COVID-19 pandemic, we continue to test these three compounds against SARS-CoV-2 (Figure 2C). Correspondingly to the activity against HAdV and RVFV, compound **1** also displayed dose–response antiviral activity against SARS-CoV-2, while compound **2** had low activity and compound **3** displayed host cell toxicity against the VeroE6 cells. Based on the results above, we selected and prioritized compound **1** for further investigations.

**Figure 3:**
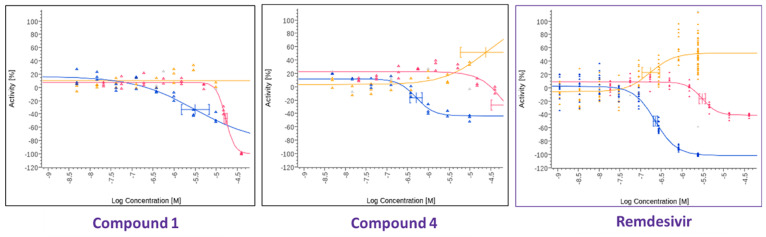
The dose–response antiviral activity of compounds **1** and **4** (Figure 1) and remdesivir as a positive control against SARS-CoV-2 in Hela3ACE2 cells. Graphs show antiviral activity measured with a SARS-CoV-2 immunofluorescence signal leading to identification of infected cells with 0% activity equals 100% infected cells (blue curve), total cells per well in SARS-CoV-2 infected cell test with 0% activity equaling no change vs. control (yellow curve), total cells per well in HeLa-ACE2 uninfected cell control (red curve).

**Figure 4:**
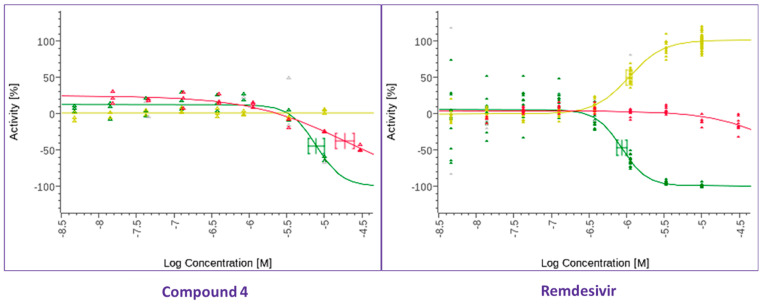
Dose–response antiviral activity against SARS-CoV-2 of compound **4** (Figure 1) and remdesivir as a positive control in Calu3 cells. The graphs show antiviral activity measured with a SARS-CoV-2 immunofluorescence signal, leading to the identification of infected cells with 0% activity equaling 100% infected cells (green curve), total cells per well in the SARS-CoV-2 infected cell test with 0% activity equaling no change vs. the control (yellow curve), and total cells per well in the HeLa-ACE2 uninfected cell control (red curve).

**Figure 5:**
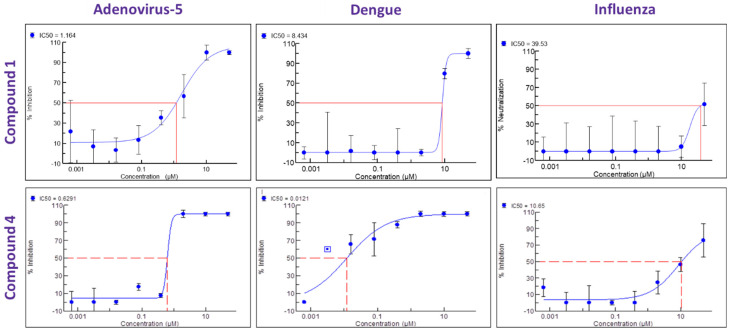
Antiviral activity dose response curves for compound **1** and compound **4** against HAdV, dengue virus, and influenza virus.

In parallel, a search was performed to identify compounds structurally similar to compound **1** within the Merck compound repository of more than one million compounds and screened an identified subset of further 25 compounds for activity against SARS-CoV-2. This resulted in the identification of compound **4** (Figure 1), with superior antiviral properties (IC_50_ = 0.5 µM, SI = 80) compared to compound **1** (IC_50_ = 3.2 µM, SI = 5), using a SARS-CoV-2/HeLa-ACE2 high-content screening assay (Figure 3). While compound **1** did not exhibit antiviral properties in a similar assay conducted with Calu-3 cells, compound **4** inhibited replication of the virus also in this cell line (IC_50_ = 7.7 µM, SI = 3) (Figure 4). We then performed a broad screening of antiviral activity against different viruses in Vero cells and, as shown in Figure 5, compound **4** potently inhibited the replication of HAdV type 5 (IC_50_ = 0.6 µM), dengue virus type 2 (IC_50_ = 0.01 µM), and influenza virus (IC_50_ = 10.7 µM) and showed dose-dependent activity against herpes simplex virus (HSV) type 2 and Zika virus (Table 1). Compound **4** shows an IC_50_ = 126 nM in the vanadate-induced inhibition of TIE-2 receptor phosphorylation in Chinese hamster ovary (CHO) cell assay. None of the compounds inhibited HSC type 1 or chikungunya virus (CHKV) (Table 1). Compound **1** also proved active against human cytomegalovirus (HCMV) (Table 1).

**Figure 6:**
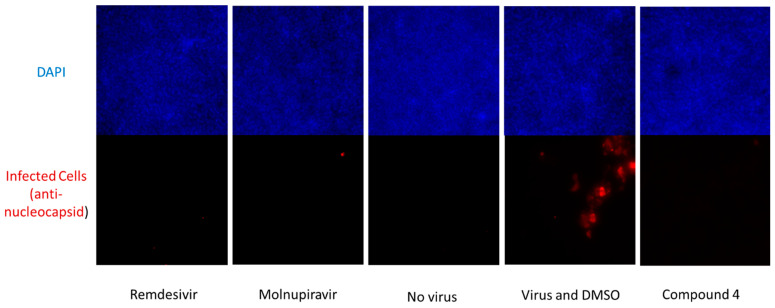
Activity in human primary mucociliary airway stem cell-based SARS-CoV-2 infection model for compound **4** compared to remdesivir and molnupiravir tested at 10 µM.

We further tested the ability of compound **4** to inhibit the replication of SARS-CoV-2 in a Primary Human Mucociliary Airway Model and, as shown in Figure 6, the compound again showed activity also in this model when tested at 10 µM (Figure 6).

**Figure 7:**
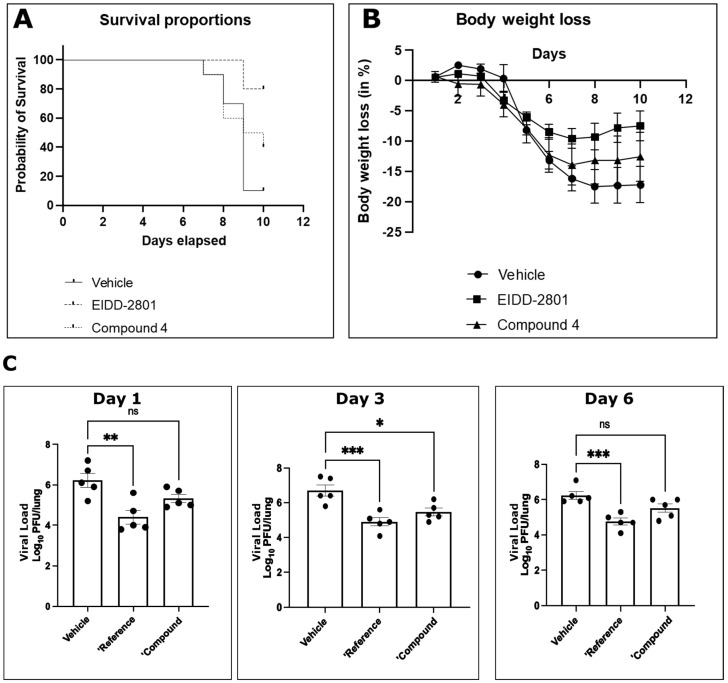
In vivo antiviral efficacy of compound **4** against SARS-CoV-2. Mice exhibited increased survival (**A**), reduced body weight loss (**B**), and reduced viral load in lungs (**C**) after oral treatment b.i.d. with 60 mg/kg compound 4 in 0.5% methocel/0.25% Tween-20/water in comparison to placebo. EIDD-2801 (molnupiravir) dosed same way was used as positive control. (ns *p* > 0.05, * *p* ≤ 0.05, ** *p* ≤ 0.01, *** *p* ≤ 0.001).

Finally, we tested for antiviral activity in vivo using a SARS-CoV-2 murine lethal challenge model (Figure 7) and chose compound **4** due to its superior antiviral profile. After oral administration, compound **4** exhibited a protective effect on animal survival in vivo after a lethal challenge (Figure 7A). Activity was also reflected by reduced body weight loss after infection (Figure 7B). Titration of viral titers in the lungs of infected mice on days 1, 3, and 6 after infection showed a statistically significant reduction in viral titers in vivo under treatment with compound **4** (Figure 7C). The effect on reduction in viral titer was comparable to the control compound molnupiravir (EIDD-2801), although the effect on survival and body weight loss was less pronounced.

The compounds were derived from a kinase inhibitor drug discovery project and in addition to TIE-2 are inhibiting further kinases. To elucidate the potential antiviral mechanism of action of this compound class, we performed a kinase inhibition profile for the superior compound **4** with DSTT (Division of Signal Transduction Therapy|University of Dundee, Dundee, UK) and Cerep (https://www.eurofinsdiscovery.com/solution/kinase-profiler, accessed on 6 March 2025). In addition to the strong inhibition of TIE-2, which was expected as the compounds were initially derived from a TIE-2 inhibitor drug discovery program, potent activity could be detected against KDR (IC_50_ = 7.9 × 10^−9^ M), CDK8 (IC_50_ = 3.1 × 10^−8^ M), MAPK14 (IC_50_ = 8.7 × 10^−8^ M), and MAPK11 (IC_50_ = 4.3 × 10^−7^ M). Involvement in viral replication has been described for MAPK14 (Banerjee, S. 2002) (Mittal, P. 2024), MAPK11 (Higgins, C. 2023), and CDK8 (Schang, L. 2002). The unique combination and simultaneous inhibition of relevant kinases makes the compound class particularly effective.

## 4. Discussion

The attrition rate for new compounds in drug discovery is high and on average only 1 out of 10 small molecules, which enter the clinical developmental phase, will finally be approved as a new therapeutic [16]. However, the access of the scientific community to such former developmental compounds is often restricted and only limited information of the available data of the (pre-) clinical development programs on these molecules are freely available. New, fresh ideas for the pharmacological use of such compounds might be obtained by investigating these compounds in as many assays and investigations as possible. Therefore, the access to those structures and the physical availability of such compounds to the scientific community allows us to assess their full potential with an option to bring these compounds back in the value chain.

In the presented work, we could demonstrate the power of open innovation and crowd-sourcing to identify new interesting leads in drug discovery, utilizing a repurposing paradigm to identify compounds with broadband antiviral activity. The identified compound class of diphenylureas constitutes an interesting starting point for further chemical modification and optimization in the frame of a lead optimization program. The fact that the compounds inhibit the replication of positive sense single-stranded RNA viruses (SARS-CoV-2, dengue virus, Zika virus), negative sense single-stranded RNA virus (influenza virus), and double-stranded DNA virus (HAdVs, HSV-2, HCMV) is promising. Interestingly, for compound **4**, the activity against dengue virus was most potent among all the viruses tested. To date, there are no approved clinically validated antivirals for the treatment of dengue virus infection and treatment remains reliant on supportive care such as fluid replacement and the use of analgesics.

For a broad-spectrum antiviral to be suitable in a clinical setting, a clean toxicology profile is of high importance and only very preliminary data are available for the compound class in that regard. The measured selectivity indices in the various cellular assays indicate a sufficient therapeutic window, and no obvious health issues were apparent in the mouse studies. In addition to the further evaluation of the potency of the chemical space via developing structure–activity relationships, future research areas may include the exploration of antiviral activity against more viral species, the evaluation of the toxicological profile of the compound class, plus elucidation of the exact mechanism of action.

Compounds **1**–**3** were derived from a program set-up to identify inhibitors of TIE-2 (tyrosine kinase with immunoglobulin-like and EFG-like domains 2), which is a receptor tyrosine kinase primarily known for its role in angiogenesis. TIE-2 has emerged as a potential target for anti-cancer therapies inhibiting angiogenesis, tumor vascularization, and tumor growth and metastasis. Interestingly, the antiviral properties for the three TIE-2 inhibitors in the Merck Mini Library varied, underlining the necessity to include structural analogs in all repurposing activities to maximize the chances of finding new therapeutic utility for existing compounds and their structure–activity relationship (SAR) environments.

Diphenylureas are important link structures in the design of an active substance for treating cancer due to their near-perfect binding with certain acceptors and have demonstrated many activities against several human cancer cell lines. Diphenylureas are utilized to treat cancer by inhibiting cell signaling transduction, such as the RAS-RAF-MEK-ERK signaling pathway and the PI3K-Akt-mTOR pathway. In addition, this structure inhibits tumor cell growth by inhibiting multiple receptor tyrosine kinases, such as Vascular Endothelial Growth Factor Receptors (VEGFRs), Platelet-Derived Growth Factor Receptors (PDGFRs), and Epidermal Growth Factor Receptors (EGFRs) [17]. TIE-2 activation has been described to protect against prothrombotic endothelial dysfunction in COVID-19 [18].

Since viruses take over a large number of host kinases at distinct steps of their life cycle, kinases represent an attractive target for broad-spectrum therapy in general, though it remains unclear what is the best combination of kinases to be inhibited. Garcia-Carceles et al. [19] mention mTOR-P13K-AKT, ABL-25, BCR/MAPK, and DNA Damage Response. Berzosertib, an inhibitor of ATR, for example, has likewise demonstrated potent activity against SARS-CoV-2 [20]. We speculate that the simultaneous inhibition of multiple cellular kinases via synergistic effects explain the in vitro antiviral properties of compound **1** and compound **4** against multiple virus families all reliant on these cellular replication pathways.

Repurposing kinase inhibitor libraries for antiviral activity offers an interesting approach to identifying compounds that could form a first line of defense in the frame of pandemic preparedness activities, as many kinases targeted in cancer also play crucial roles in viral replication and immune response modulation. Kinase inhibitors, by targeting host cell pathways, may offer a broad-spectrum antiviral potential, potentially reducing the likelihood of viral resistance formation compared to antivirals that are directly acting on viral proteins. Positive examples have already been published such as, e.g., Garcia, G. et al. 2021, Perwitasari et al. 2015, Schor 2018, Weisberg et al. 2020, Zhang, D. et al., El Bairi et al. 2020 or Naik, R. et al., 2022 [21,22,23,24,25,26]. Which kinases exactly to inhibit for optimal broad-spectrum antiviral activity will require substantial further investigations of the scientific community and no clear consensus has been established yet. For compound **4**, a preliminary kinase inhibition profile was performed. In addition to the strong inhibition of TIE-2, which was expected as the compounds were initially derived from a TIE-2 inhibitor drug discovery program, potent activity could be detected against KDR, MAPK14, CDK8, and MAPK11. Involvement in viral replication has been described for MAPK14 [27,28], MAPK11 [29], and CDK8 [30]. The mechanism of action is therefore likely due to a combined inhibition of MAPK14, MAPK11, and CDK8, potentially enhanced by the inhibition of other targets relevant for the replication of various viral families. The dependence of different viruses on various kinases varies with cell type and physiological status and the overlap of target hits in corresponding genome wide knock-out or knock-down screens has only been marginal [31].

The antiviral profile of the compound class is very interesting, covering a whole range of viruses causing a high disease burden such as SARS-CoV-2, adenovirus, and influenza. Compound **4** interestingly shows a very high potency against dengue virus (IC_50_ = 0.01 µM), a pathogen that causes rising global concern [32]. Dengue virus is now a major global health challenge, infecting an estimated 390 million people annually in over 100 countries. The increasing incidence, expanding geographic range, and the potential for severe disease (Dengue Hemorrhagic Fever and Dengue Shock Syndrome) underscore the urgent medical need for effective antivirals, as currently no approved antiviral drug exists for dengue.

Overall, integrating kinase inhibitors into pandemic preparedness could enhance our ability to combat emerging viral threats by targeting the conserved host pathways required for replication instead of the highly variable viral proteins themselves. For this activity to be successful, it is of utmost importance to compile a library of promising compounds that already exist and that can quickly be profiled with high probability of success against newly emerging so far unknown pathogens. The day will come when we need them.

## Figures and Tables

**Table 1:** Overview of antiviral activity of compound **1** and compound **4** against different viral species with disease relevance.

IC50 [µM]	SARS-CoV-2	HAdV	CHKV	DENV	HSV-1	HSV-2	INFV	ZIKV	HCMV
Compound 1	3.2	1.16	Not Active	8.43	Not Active	Active	39.5	Not Active	Active
Compound 4	0.5	0.63	Not Active	0.012	Not Active	Active	10.7	Active	Not tested

## Data Availability

Data is contained within the article.

## Appendix A

**Table A1:** Target classes of compounds included in the Merck Mini Library.

Target	No. of Cmpds
AT1R blockade	5
Ca(2+) sensitization (of myofibrillar proteins)	4
(Peripheral) D2-, 5HT1a-, α1-, α2-agonism	1
(Presynaptic) D2-agonism, 5HT reuptake	
inhibition, 5HT1A agonism	1
(Peripheral) D2-agonism	1
Sigma ligand, functional D2-antagonism	1
DP4 -antagonism	1
DHODH inhibition	1
DP2/CRTH2 antagonism	4
EP2-/EP4 inhibition	4
ETA-R blockade	3
GLEPP1 inhibition	3
IKK2 inhibition	1
LAP-R blockade	2
MetAP2 inhibition	5
MPP12 inhibition	2
NHE1 inhibition	6
K(ATP) potassium channel opening	5
PDE3 inhibition (Isomazol)	6
PDE3 inhibition (Meribendan)	6
PDE4 inhibition	4
PDE5 inhibition	4
PI3Kgamma inhibition	5
SPHK1 inhibition	3
TIE-2 inhibition	3
**Total:**	80
